# Cognitive neuroepigenetics: the next evolution in our understanding of the molecular mechanisms underlying learning and memory?

**DOI:** 10.1038/npjscilearn.2016.14

**Published:** 2016-07-20

**Authors:** Paul Marshall, Timothy W Bredy

**Affiliations:** 1 Department of Neurobiology and Behavior and Center for the Neurobiology of Learning and Memory, University of California Irvine, Irvine, CA, USA; 2 Queensland Brain Institute, The University of Queensland, Brisbane, QLD, Australia

## Abstract

A complete understanding of the fundamental mechanisms of learning and memory continues to elude neuroscientists. Although many important discoveries have been made, the question of how memories are encoded and maintained at the molecular level remains. So far, this issue has been framed within the context of one of the most dominant concepts in molecular biology, the central dogma, and the result has been a protein-centric view of memory. Here, we discuss the evidence supporting a role for neuroepigenetic mechanisms, which constitute dynamic and reversible, state-dependent modifications at all levels of control over cellular function, and their role in learning and memory. This neuroepigenetic view suggests that DNA, RNA and protein each influence one another to produce a holistic cellular state that contributes to the formation and maintenance of memory, and predicts a parallel and distributed system for the consolidation, storage and retrieval of the engram.

## Introduction

Learning is described as a persistent, experience-dependent change in behaviour and memory as the internal representation of this experience, which has traditionally been defined as the engram.^1–3^ How organisms learn has been a question of interest since before the days of Darwin, who proposed that organisms gain innate adaptation through evolution, or Lamarck, who argued that this must occur in response to current environmental demand and is therefore acquired in a lifetime.^4,5^ The importance of this question is further echoed by those who have addressed it across a variety of levels of analysis, including animal behavior,^6^ cognition,^7,8^ development^9^ and the physiology underlying synaptic transmission, each domain providing its own important contributions, as well as caveats.^10^

Most empirical evidence suggests that memory formation has two primary components, one that is protein-synthesis independent, and a second time-dependent phase that relies on activity-induced gene transcription and protein synthesis, which lead to enhanced synaptic efficacy.^10,11^ This is based on the observation that protein synthesis occurs in a predictable time frame following a behavioural experience through a process known as memory consolidation, and that protein-synthesis inhibitors, when administered within this period, block the formation of memory.^2,3^ However, several reports call into question the strength of this perspective, including (1) the non-specific effects of protein-synthesis inhibitors (e.g., anisomycin can also influence neurotransmitter release, and its effects can be rescued without affecting protein synthesis *per se*).^12–14^ Protein-synthesis inhibitors can also induce phosphorylation of CREB^15^ and apoptosis;^16^ (2) the demonstration of memory formation in a variety of tasks even during more than 90% reduction of protein;^17–20^ (3) the existence of simple and selective forms of cellular memory that are protein-independent, such as protection from viral integration in plants,^21^ and (4) the fact that it is implicit in many empirical descriptions of this phenomenon that protein synthesis is sufficient for long-term memory.

It is evident that we are still a long way from fully understanding the intimate relationship between protein synthesis and memory, and at the very least questioning of the sufficiency, but not necessity of protein synthesis may be beneficial. In fact, a re-evaluation of the protein-synthesis hypothesis of memory may open up new opportunities for understanding, such as determining whether memory is established by serial or parallel processes, as well as questions about the location of the engram.^22^

The general way we think about memory derives from William James’ distinction between sensory processing (primary memory), and a more permanent trace of this processing (memory proper), that is intuitively linear if we think about storage.^23^ Unfortunately, this linear trajectory does not follow for less well-known memory functions, such as re-updating and retrieval.^24,25^ In addition, we know from the seminal work of Brenda Milner with patient H.M. who suffered severe memory impairment following a bilateral temporal lobectomy, that sensory processing and storage of experience can be dissociated for particular types of memories, presumably by disrupting this linear sequence. Much like early observations on the use of protein-synthesis inhibitors in memory, this work demonstrated that the hippocampus is necessary, but not sufficient, for all memory.^26^ These findings elicited further work, which suggested that particular types of memories are dependent on certain regions of the brain^27–29^ but also rely on parallel communication with other regions, in some cases competitively,^30,31^ leading to the conclusion that no one region is sufficient to consolidate, store and retrieve all memory. In comparison, if one assumes that protein synthesis is sufficient for memory, it naturally follows that memory must follow a serial path that terminates in the production of protein. This view derives from the central dogma, which dominated molecular biology for many years and proposed a linear trajectory from DNA to RNA to protein. This view is so entrenched in molecular biology that Crick himself put out an explanatory paper describing how his work had often been misinterpreted as an oversimplification of multiple possibilities. However, despite this, it has remained as an implicit bias in molecular neuroscience.^32^ Thus given the questioning of a serial view of memory at the molecular and cognitive levels, an alternative and parallel view warrants exploration.

By the same logic, protein sufficiency and its consequences for the location of information storage can be questioned. Following a protein-centric view, the end goal of all molecular changes is primarily a change in protein level, and the structural and change in synaptic efficacy that follows must serve a general storage function. This is akin to Lashley’s equipotentiality argument, which stated that one brain region could serve the same general function as any other for memory.^22^ However, this idea of systems equipotentiality was challenged long ago by the discovery of discrete brain regions for language production and understanding, such as Broca’s and Wernike’s areas,^33^ although we have not questioned this at the molecular level.

**Figure 1:**
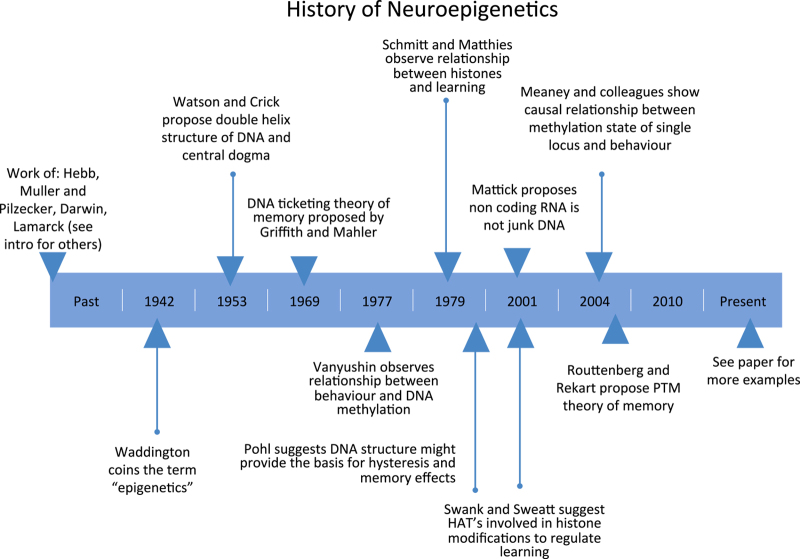
Timeline of significant discoveries in the field of cognitive neuroepigenetics.

An emerging, and potentially complementary, view is that neuroepigenetic mechanisms, which constitute bidirectional and reversible changes in nucleic acids and proteins, occur both before and after transcription. These effects proceed within the same time frame as learning, and therefore represent an attractive alternative to the extreme interpretations of adaptation and heritability by Lamarck and Darwin.^34^ Contrary to contemporary perspectives, the concept of epigenetics within the context of learning and memory, or cognitive neuroepigenetics as it is now known, is not new (Figure 1). For example, more than 40 years ago, Griffith and Mahler^35^ proposed the DNA ticketing theory of memory, which postulated that the source for memory lay in the modification of nucleic acids, and suggested that the engram could extend beyond changes in neuronal function that are the result of protein synthesis alone.

Although this model was not directly testable due to technical limitations at the time, certain predictions have held true. For example, Vanyushin provided early experimental evidence that DNA methylation is associated with both active avoidance and food-seeking in rats.^36^ These pioneering findings were later rediscovered by Sweatt and colleagues, who showed an association between gene-specific DNA methylation, histone modification and memory formation.^18–21^ These studies have led to other intriguing advances, including the demonstration that molecular substrates classically associated with learning and memory, such as CREB-binding protein, also possess histone acetyltransferase activity, which accounts, in part, for some of their ability to modulate memory.^37,38^ Thus, the field has been primed to interrogate epigenetic mechanisms of DNA methylation and histone acetylation to establish their generality, and elucidate their complementary effects.

Meaney and colleagues discovered a causal role for epigenetic mechanisms in behavioural regulation by showing that variations in maternal care lead to reversible changes in DNA methylation within the glucocorticoid receptor promoter, resulting in significant effects on reactivity to stress later in life.^39,40^ This seminal discovery set the stage for the investigation of new epigenetic marks that are both behaviourally induced, and gene locus-specific. For example, Kumar *et al.*^41^ found that histone modification is associated with the progression from acute to chronic drug use, whereas Bredy *et al.*^42^ provided evidence to suggest that patterns of histone acetylation around a single gene promoter can be influenced in different ways depending on the type of learning. Many of these initial studies have been overlooked based on the fact that neuroepigenetic mechanisms initially did not appear to be persistent, and thus could not serve a role in maintaining the memory trace. This issue has been discussed by pioneers in the field, including Crick,^43^ Lisman,^44^ and Sweatt,^45^ as a key criterion for any molecular substrate to be affiliated with molecular memory processes. However, more recent studies have reported long-term persistent changes in epigenetic mechanisms, as well as demonstrating that some initial epigenetic marks may transition to others.^46,47^

Taken together, the findings suggest that neuroepigenetic mechanisms could provide a foundation for testable hypotheses of changes on nucleic acids as well as protein in relationship with a memory code.^27–29^ In addition, the strength of a neuroepigenetic view of learning and memory is that it diverges from traditional molecular neuroscience, which itself stems from the central dogma of molecular biology.^28^ This bias within molecular biology has most notably led to the labelling of non-coding regions of DNA as genetic *noise* or ‘junk DNA’, a conclusion that has since proven to be incorrect.^48^ As an extension of this, we argue that the unstated assumption of protein as the final goal of transcription has led to just as critical an oversight in the search for the fundamental molecular mechanisms of learning and memory, and that neuroepigenetic mechanisms offer an alternative explanation of the molecular underpinnings that lead to the engram, which are bidirectional, parallel and mechanistically dispersed.

## Influence of DNA: old player, new roles in the adult brain?

### DNA modification

There are a variety of DNA modifications; however, relatively few have been studied in the context of learning and memory. So far, the canonical modification, 5-methylcytosine (5mC) has mostly been associated with gene repression.^49^ However, the accumulation of 5mC is dynamic and has also been shown to enhance gene expression. Therefore, it has a far more functionally relevant role in the regulation of activity-dependent gene expression than previously assumed.^50^ This epigenetic mark also has oxidative derivatives, including 5-hydroxymethylation (5hmC), which has been shown to regulate gene expression within the context of learning and memory.^47,51^ Moreover, a recent study has demonstrated that the DNA glycosylases Ogg1 and MutY, which target the base modification 8-oxoG, have a role in adaptive behaviour, which implies a physiologically relevant role for 8-oxoG in the adult brain.^52^ Further, in a series of preliminary experiments, we have discovered that the accumulation of N6-methyladenosine (m6A) on DNA increases following extinction learning, and that knockdown of the putative m6A methyltransferase N6AMT1 blocks the consolidation of extinction memory (Xiang Li *et al.*, unpublished). Contrary to early studies showing an inverse relationship between 5mC and gene expression in the brain, we found that the accumulation of m6A is required for the recruitment of the transcriptional machinery and serves to drive activity-dependent gene expression. In addition, emerging biochemical evidence suggests that there are many modified bases beyond cytosine that are theoretically functional.^53,54^ Mutagenesis and epileptogenesis studies have also shown that some of these base modifications can be induced under physiologically relevant conditions, similar to the way in which some histone modifications were recognized.^54–56^ These findings suggest the existence of a diverse and potentially functional repertoire of reversible DNA modifications on all four bases that could contribute to learning and memory processes.

### DNA structure

It is often assumed that DNA only encodes information in its nucleotide sequence. This is partly because when Watson and Crick proposed their double-helix model of DNA they described a right handed form of DNA now called B-DNA, and implied a static conformation. However, Pohl^57^ found that changes in the conformational state of DNA can be detected based on previous changes in DNA structure. This not only suggests that DNA structure influences downstream effects such as protein binding, but also indicates that there is a molecular representation of a previous experience that can persist over time and is reflected in the current conformation of DNA. Pohl therefore suggested that, ‘DNA might provide the basis for hysteresis and memory effects…[and thus] should at least be considered as a possibility in biological systems.’ Indeed, much like its RNA and protein products, DNA can encode information through dynamic changes in its secondary structure.^58^ In fact, DNA can adopt at least 20 different conformations, which can act in concert with or independently of the sequence to regulate, among other functions, the recognition specificity of binding proteins.^41–44^

There also appears to be an interaction between DNA modification and structure, as specific conformations can be recognized by DNA-modifying enzymes, and changes in structure can modulate the ability of these enzymes to modify DNA.^59,60^ Furthermore, chromatin states can also interact and be influenced by DNA structure.^61^ Despite this, the prevailing view is that changes in DNA structure likely represent transcriptional and translational noise and are the by-product of transcription, adopted only in the absence of chromatin compaction during nucleosome remodelling.^62^ However, based on the current evidence, it is plausible that there are both stable and transient DNA conformational changes. These conformational changes may occur briefly as a result of transcription or local cellular state change, or be bound by base modifications and potentially rendered stable. If confirmed, this would represent yet another example of a parallel and bidirectional mechanism distributed across the genome, which may encode part of the engram.

### DNA editing

The possibility of DNA editing is contentious in light of the current conceptualizations of the genome and its functional readout, in large part because stability is assumed. DNA editing refers to any collection of mechanisms that are predictably engaged to modify the underlying sequence of DNA in an experience-dependent manner. The most well characterized example of this can be found in the immune response, which is an exquisite memory system. However, beyond the variable diversity joining (VDJ) recombination process and somatic hypermutation associated with diversification of the immune system, there are emerging reports of other forms of functionally relevant DNA editing, including retrotransposon insertion and dynamic single-nucleotide variants (SNVs), which can occur anywhere in the neuronal genome. Although it was previously assumed that retrotransposition only occurred during early embryogenesis,^63^ it has now been demonstrated that the expression of the long interspersed nuclear element 1 (L1) retrotransposon continues during adulthood and is elevated in the brain.^64^ L1 retrotransposition occurs in response to a range of environmental stimuli, including voluntary exercise and chronic cocaine exposure.^65,66^ Indeed, single-cell retrotransposon sequencing analysis has recently revealed pervasive L1 mobilization in human hippocampal neurons.^67^

With respect to SNVs, it is becoming increasingly evident that each post-mitotic neuron in the human cortex can have a distinct genome, with conservative estimates of around 1,500 somatic SNVs per neuron,^68^ whereas others suggest that up to 10,000 SNVs may accumulate in healthy differentiated neurons across the lifespan.^69^ Pena de Ortiz and colleagues have for many years reported on the profound DNA recombinase activity that occurs in the brain in response to experience.^70–72^ Finally, DNA double-strand breaks have recently been shown to be necessary to regulate the expression of immediate early response genes, which are known to be important for learning and memory.^73^ Together, the evidence suggests that DNA editing may serve as a critically important source of functional diversification in post-mitotic neurons, enabling them to optimize their transcriptional responses to rapidly changing environmental signals by destabilizing and actively changing underlying genomic code. This is a significant departure from the concept of DNA as a static carrier of heritable information; however, its role in the regulation of gene expression related to learning and memory remains to be explored further.

## Influence of RNA: ancient mechanisms, new neuroepigenetic player?

Despite possessing superior cognitive processes, ‘higher order’ organisms share approximately the same number of protein-coding genes with lower eukaryotes. Non-coding DNA-derived transcripts that possess no protein-coding capacity (non-coding RNA) have instead increased in the mammalian genome across evolution.^48^ Various classes of non-coding RNAs have been shown to participate as modular scaffolds and decoys, in cellular localization, and importantly, in activity-dependent cellular processes independent of protein, such as protection from viral infection.^21,74,75^ Recent evidence also indicates that different classes of non-coding RNA are directly involved in learning and memory. For example, microRNAs are critically involved in various forms of fear-related learning and memory,^76,77^ and recent studies have shown that long non-coding RNAs are also regulated by experience and appear to play a role in behavioural adaptation.^78,79^ Moreover, much like DNA, the post-transcriptional regulation of RNA is influenced by dynamic changes in chemical modification, structure, and editing.

### RNA modifications

One recently emerging mechanism for how RNA is epigenetically regulated is through chemical modification. To date, there are at least 140 ‘epitranscriptomic’ modifications that are known to occur in RNA. Although little is currently known about their function in the context of learning and memory,^80^ one can postulate about their role. Pseudouridine (pseudoU) has been shown to affect RNA decay, potentially maintaining RNA involved in a short-term trace, or prolonging that associated with the long-term processes of memory consolidation.^81,82^ In addition, an enzyme which promotes the accumulation of pseudoU has recently been shown to be associated with cognitive dysfunction.^83^ These chemical modifications are also targeted to RNA via small nucleolar RNAs, which themselves have been shown to be involved in behavioural adaptation.^84,85^ RNA modifications have been demonstrated to affect the qualitative nature of RNA translation, thus constituting yet another potential way to alter protein function in an activity- or experience-dependent manner.^86,87^ In recent work, we have discovered that the RNA modification m6A is highly dynamic in the brain and critically involved in the formation of fear memory.^88^ We predict that, like DNA modification, RNA modification will come to be appreciated as an important epigenetic mechanism associated with behavioural adaptation that occurs both bidirectionally and in parallel to other neuroepigenetic mechanisms.

### RNA structure

Chemical modifications are also known to affect the folding of RNA and are critical for determining its secondary and tertiary structures, which can impact the function of RNA inside the cell.^74,89^ This is interesting because changes in RNA structure have been linked to learning and memory. A stem-loop structure in the 3′ untranslated region of brain-derived neurotrophic factor (BDNF), a key neurotrophic factor for learning and memory, has been shown to be calcium-dependent and necessary for RNA stabilization.^90^ Furthermore, the G-quadruplex RNA structure is required for calcium/calmodulin-dependent protein kinase type II alpha chain and postsynaptic density protein 95 localization to neurites, both key factors in plasticity required for learning.^91^ It has also been shown that alternative splicing of exon 10 of tau, a protein that is strongly linked to neurodegenerative processes that lead to deficits in learning and memory, is regulated by a stem loop induced by a particular RNA helicase.^92^ Thus, dynamic changes in RNA structure may represent a novel mechanism for how RNA is co-opted for memory processes, without the need for new protein synthesis.

### RNA editing

RNA editing is a process whereby an organism can increase the complexity and repertoire of transcripts that are able to be produced without changes in the genetic code.^48,93^ Specifically, two major classes of enzymes, including the ADAR family of adenosine deaminases and the APOBEC family of cytosine deaminases, mediate RNA editing. In what are now classic examples of the functional relevance of RNA editing, ADAR1 and two have been shown to promote the editing of the 5-hydroxytryptamine 2c receptor and the GluR2 subunit of the AMPA receptor, and can even modify synaptic structure, all of which are known to affect learning and memory.^94^ In addition, other RNA editing enzymes such as ADAR3 are only expressed in the brain of higher order vertebrates, which further suggests unexplored roles for RNA editing in cognition.^95,96^ It has also been shown that RNA editing can be altered by RNA structure, suggesting that RNA editing might represent a complementary and parallel process that can act on the qualitative state of a protein but is dependent on other protein synthesis-independent pathways in the cell.^97^

## Influence of protein: old player, new tune

The conceptualization of protein synthesis-dependent learning and memory dates back to some of the first studies performed to understand biological processes contributing to memory, in which ‘protein-synthesis inhibitors’ were used to demonstrate the necessity of protein for memory storage (reviewed in refs 2,3). Unfortunately, as outlined above, these drugs have been shown to act by impairing nascent RNA, as well as a plethora of off-target processes, including phosphorylation of CREB, modifying adrenergic release, and potentially generating state dependency, all of which complicate the interpretation of their effect.^12–14,98,99^ However, classic examples of the necessity of protein synthesis for memory should not be overlooked. These include many different examples involving a variety of intracellular signalling cascades, as outlined by Kandel and others, including simple habituation in *Aplysia*, as well as fear learning in mice.^11^ In addition, histone modifications seem to act in part by altering gene expression and protein levels.^100^ Moreover, another way in which proteins may overcome molecular turnover is via prion-like conformational state changes, which can by definition self-perpetuate.^101^ Similarly, Routtenberg and Rekart^102^ have proposed the post-translational modification of protein hypothesis, which states that instead of relying only on static protein machinery for the formation and maintenance of a memory, memory can also be quasi-stored in dynamic, but consistently similar, post-translational modifications of proteins, which can affect subsequent processing of stimuli without the need to be in an active state. This idea is intriguing, and has been expanded within this paper to include DNA and RNA alongside traditional protein-synthesis views.

## Neuroepigenetics: dynamic integration across all levels

**Figure 2:**
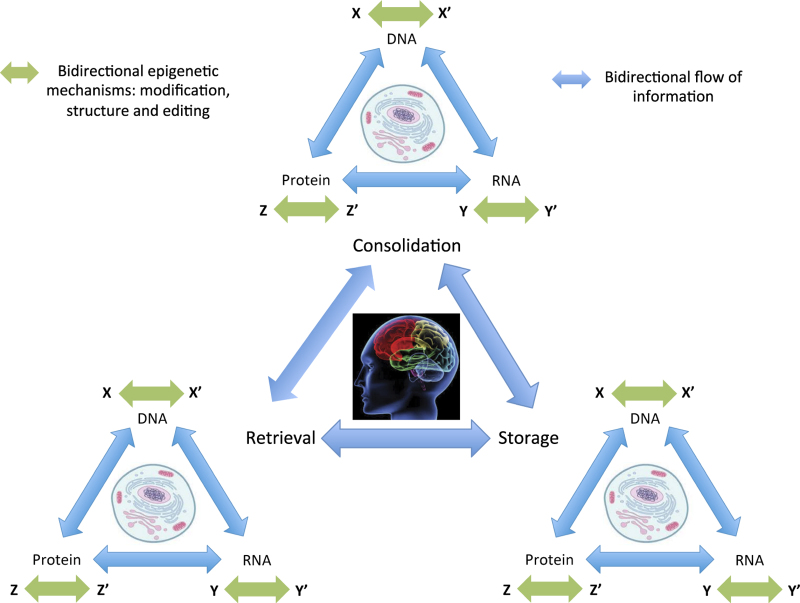
A neuroepigenetic model of memory suggests dynamic and reversible, state-dependent, modifications on DNA, RNA and protein that occur during consolidation, storage and retrieval. Much like Roberson and Sweatt’s^45^ model: X indicates cytosine and X′ 5-mC, Y adenosine and Y′ N6-methyladenosine, Z is acetylated histone and Z′ deacetylated histone. Although more evidence is required to establish the generalized nature of this model across all phases of learning and memory, it is well established that epigenetic mechanisms influence memory storage, and emerging findings suggest an influence on consolidation^102^ and retrieval. ^104–106,^^111–123^

It is likely that the mnemonic state of an organism is encoded by changes in both nucleic acids and proteins, which can be temporarily biased or bounded by modifications, editing and structural changes to produce memory. Coming back to the question of serial or parallel systems and the location of memory storage, we can integrate what is known about protein synthesis, post-translational modifications and neuroepigenetic mechanisms into a more holistic view of the molecular basis of learning and memory (Figure 2).

As mentioned above, the process of encoding and memory storage is intuitively linear. However, when speaking about memory it is important to keep in mind that, much like the fact that transcription does not always lead to protein expression, encoding does not always result in storage, which in turn does not always lead to retrieval. Memories can be state-dependent, inaccessible at one point in time but, with a change of internal state, immediately retrieved. For example, Gisquet-verrier *et al.*^103^ have shown that impairment by protein-synthesis inhibitors can be reversed if they are delivered both post-training and pre-test. Is this because of alterations to existing proteins or has the epigenetic state of DNA, RNA and protein been changed? The evidence remains to be seen, but the question is important. Put more simply, experience could be encoded across a spectrum of epigenetic modifications to DNA, RNA and protein, and over time this reversibility may become less probable and a similar cellular state may be more easily reproducible, such as occurs in the case of metaplasticity and epigenetic priming. The strength of this complementary view is that this molecular system is parallel and distributed, instead of serial and equipotential, which is also in line with the emerging evidence in neuroepigenetics. This view also leads to the prediction that most memories will be metastable and cannot by definition be an exact replication at the molecular or cognitive level of the past; instances when this does occur would necessitate moving a chemical equilibrium so far in one direction that its reversibility becomes biochemically unfeasible, including the chemical stability of protein formation.

## What is on the horizon?

The question of where cognitive neuroepigenetics as a field is going requires an appreciation of the precision of emerging technology and clarity in how current technologies are applied. Currently, we know that there are a variety of ways in which nucleic acid can alter memory and learning, but there is a paucity of data to definitively link all of the pieces. More explicitly, there are three requirements that need to be met in order to tackle this issue: (1) specific measurement, (2) specific manipulation and (3) functional validation. Specific measurement refers to the need to extend fundamental data about established brain regions and molecular pathways, based on cell-type and locus-specific analyses. A promising approach to achieve this resolution is single-cell sequencing, which has already begun to reveal a level of precision often missed in population studies.^68^ In addition, we have developed methodology to enrich and profile epigenetic mechanisms in specific cell types, including those that are selectively involved in a memory trace.^104^ Furthermore, others have developed ‘click DNA labelling’, which uses chemically specific interactions with selected targets such as DNA modification, to produce genome-wide profiling of this epigenetic mark at single base resolution.^105,106^ Together, these technologies can be applied to the analysis of particular active cells, loci and nucleotide modifications that potentially make up Lashley’s dynamic engram at the molecular level.^22^

With an exceptional level of detail in the measurement of epigenetic changes on the horizon, this begs the question as to what is required to best use the information generated from these studies. Thankfully, there already exists a technique to locus-specifically edit and direct putative modifications in the brain, namely clustered regularly interspaced palindromic repeat (CRISPR)-Cas9. This new technology uses the enzymatic machinery from bacteria, which normally functions to target and destroy viruses, and instead exchanges the RNA targeting sequence to virus for one of an experimenter’s choice to selectively target regions of DNA for excision.^107,108^ Furthermore, and more excitingly for the field of neuroepigenetics, this technique can also use a deactivated version of the cutting enzyme (dCas9) to site-selectively direct proteins, or epigenetic modifiers in awake behaving animals to probe learning and memory processes. For example Heller *et al.*^109^ have used a similar technology called transcription activator-like effector nuclease (TALEN), which uses a cutting enzyme and direction sequence, to functionally validate the sufficiency of modifications around one locus to modulate a behavioural effect. This technique has also been paired with light activation to reversibly modulate epigenetic modifiers *in vivo* in a rapid and discrete temporal window.^110^ In the future, these kinds of studies should serve as the standard. In particular, as techniques for measurement and manipulation evolve, so too should it become less acceptable to simply relate bulk protein or mRNA levels to behavioural change, as the data no longer support the 1:1 relationship as outlined in central dogma.

## Conclusions

Sufficient evidence now exists to support the concept of reversibility as a common thread in our understanding of the molecular mechanisms of learning and memory, which extends well beyond the traditional protein-centric model. It is evident that nucleic acids and related epigenetic mechanisms contribute to learning and memory in a variety of ways, and can bidirectionally impact each stage of the cognitive process. A neuroepigenetic view predicts a parallel and distributed system for the consolidation, storage and retrieval of the engram based on dynamic and reversible changes to DNA, RNA and protein in the brain. This view may also help to explain the increased complexity of higher order organisms and how they have evolved to maintain their capacity to learn and store information despite constant changes in the environment.
